# Molecular Mechanisms Underlying Vertebrate Adaptive Evolution: A Systematic Review

**DOI:** 10.3390/genes14020416

**Published:** 2023-02-05

**Authors:** Francelly Martínez Sosa, Małgorzata Pilot

**Affiliations:** 1 Museum and Institute of Zoology, Polish Academy of Sciences, 80-680 Gdańsk, Poland; 2 Faculty of Biology, University of Gdańsk, 80-308 Gdańsk, Poland

**Keywords:** adaptive evolution, vertebrates, regulatory mechanisms, environmental adaptations, gene loss

## Abstract

Adaptive evolution is a process in which variation that confers an evolutionary advantage in a specific environmental context arises and is propagated through a population. When investigating this process, researchers have mainly focused on describing advantageous phenotypes or putative advantageous genotypes. A recent increase in molecular data accessibility and technological advances has allowed researchers to go beyond description and to make inferences about the mechanisms underlying adaptive evolution. In this systematic review, we discuss articles from 2016 to 2022 that investigated or reviewed the molecular mechanisms underlying adaptive evolution in vertebrates in response to environmental variation. Regulatory elements within the genome and regulatory proteins involved in either gene expression or cellular pathways have been shown to play key roles in adaptive evolution in response to most of the discussed environmental factors. Gene losses were suggested to be associated with an adaptive response in some contexts. Future adaptive evolution research could benefit from more investigations focused on noncoding regions of the genome, gene regulation mechanisms, and gene losses potentially yielding advantageous phenotypes. Investigating how novel advantageous genotypes are conserved could also contribute to our knowledge of adaptive evolution.

## 1. Introduction

Vertebrates colonized a broad range of habitats with varying ecological conditions (e.g., lighting conditions, oxygen content, and water salinity). This process was associated with adaptations to these varied environments. Adaptive evolution is a process by which advantageous phenotypes, i.e., traits which increase fitness, are propagated through positive selection or environmentally induced variation [1]. Advantageous traits are determined by evolutionary pressures and habitat dynamics; consequently, a phenotype that is advantageous in certain ecological contexts might be neutral or deleterious in others. 

**Figure 1:**
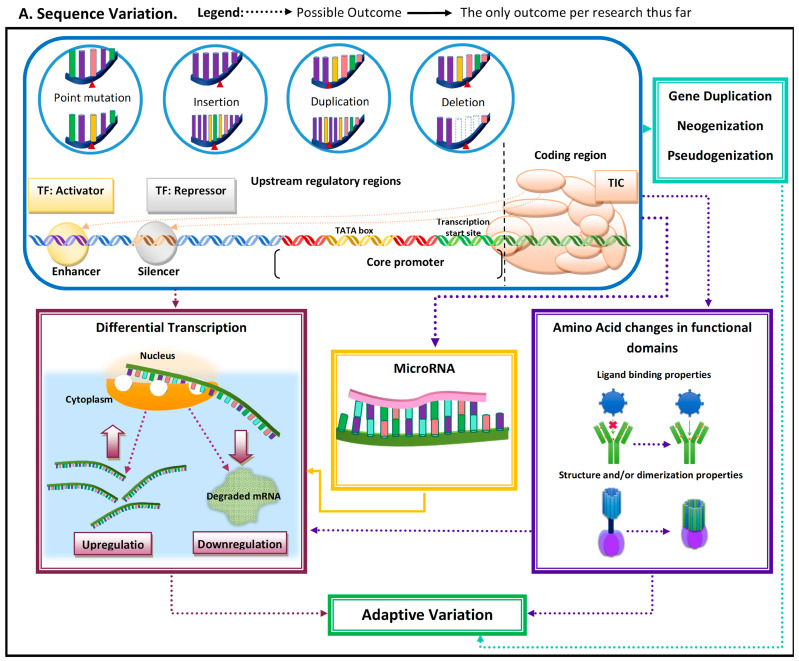

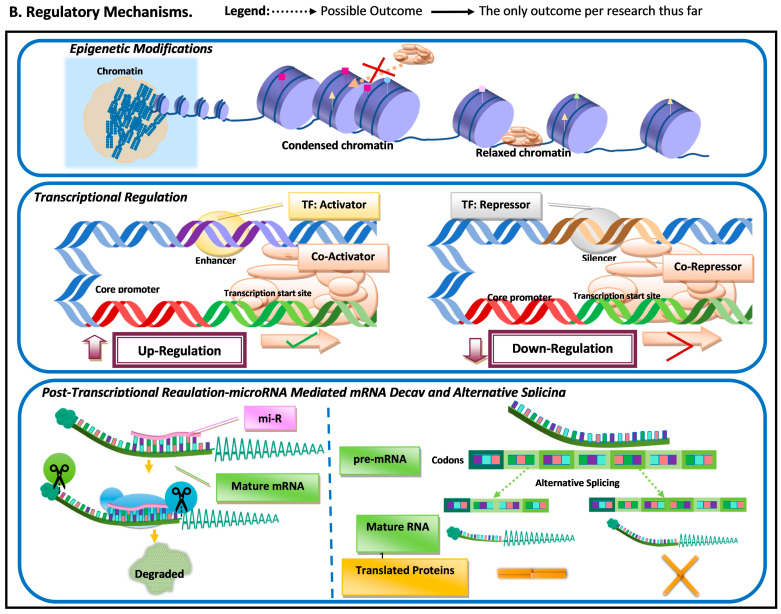
Schematic representation of the most common molecular mechanisms underlying adaptive variation in vertebrates. (**A**) Sequence variation. Sequence variation refers to any change in DNA sequence that commonly occurs due to DNA polymerase errors (e.g., slippage and erroneous excisions) and/or insertion or deletion of transposable elements. DNA sequence variation in conserved elements (i.e., regulatory elements and coding regions) is the most common mechanism underlying adaptive variation in vertebrates and can lead to gene duplication, neogenization, and pseudogenization. Sequence variation in coding regions can lead to amino acid changes at or near functional domains, which can impact protein function and/or structure. Additionally, sequence variation at the coding regions can also lead to the emergence of microRNA (miR) or modification in existing miRs. MiRs can hybridize with target mRNA and can lead to its decay, thus downregulating gene expression. Sequence variation in regulatory elements can lead to increases (upregulation) or decreases (downregulation) in transcription of a gene. (**B**). Regulatory mechanisms. The three most common regulatory mechanisms underlying adaptive variation in vertebrates described thus far are epigenetic modifications, transcriptional regulation, and post-transcriptional regulation. Epigenetic modifications are modifications to histones, the protein component of nucleosomes. These modifications can impact an RNA polymerase’s ability to find core promoters as well the activity of transcription factors. Transcriptional regulation involves cis-regulatory elements and trans-regulatory elements, which impact the function of the transcription initiation complex (TIC). Post-transcriptional regulation involves elements that impact the messenger RNA transcript itself. One of these mechanisms is microRNA (mi-R)-mediated mRNA decay, a process that leads to the degradation and subsequent downregulation of said transcript. Another mechanism that can impact the translated protein structure and/or function is alternative splicing.

Adaptive evolutionary processes occur in two general steps: (1) variation arises and (2) advantageous phenotype is propagated and maintained in the population. Genetic variation arises from mutations that can occur due to single base changes, insertions, deletions, and duplications [2]. Mutations can have multiple implications, from pseudogenization to de novo emergence of genes and/or regulatory elements (Figure 1 and Table 1). Mutations can give rise to novel phenotypes, and the advantageous ones become propagated in a population [2]. Thus, functionally important genomic regions are assumed to be highly conserved and adaptive alleles undergo rapid fixation [3,4]. The conserved nature of these functionally important elements can be used to identify them as being adaptive. 

As novel approaches continue to emerge, we are now able not only to identify positive selection on genomic regions but also to investigate the molecular mechanisms underlying adaptive phenotypes. The aim of this systematic review is to discuss specific examples of molecular mechanisms driving adaptive evolution in vertebrates in response to ecological factors, such as differences in lighting conditions, aquatic and terrestrial environments, extreme conditions, diet diversification, pathogens, reproductive adaptations, and others. We focus here on recent studies on adaptive evolution (from 2016 to 2022), which were not covered by earlier reviews on this topic [1,2]. A more recent review [5] was focused specifically on adaptive convergent evolution, while the current review has a broader scope, providing descriptions of the molecular mechanisms involved in the emergence of adaptive variation.

## 2. Materials and Methods: Evidence Acquisition

The database Web of Science was searched using the following Boolean string: “(“adaptive evolution” or “adaptive evolution mechanism$” or “adaptive evolutionary mechanism$” or “molecular adaptive evolution” or “transgenerational adaptation” or “adaptat$ epigenetic” or “adaptat$ epigenetics” or “local adapatation$”) AND (“mammal$” or “vertebrat$” or “fish$” or “bird$” or “reptile$” or “amphibian$” or “avian reptile$” or “non avian reptile$”) NOT (plant$ or invertebrate$ or fung$ or drosophila or “information technology”)”. To address a concern of one of the study reviewers that studies on reproductive adaptations are underrepresented in this review, we carried out an additional search specifically focused on this topic, using the following query: “(“reproduction or reproductive”) AND (“adaptive evolution” or “molecular adaptation”) AND (“mechanism” or “epigenetic” or “regulatory”) AND (“mammal$” or “vertebrat$” or “fish$” or “bird$” or “reptile$” or “amphibian$” or “avian reptile$” or “non avian reptile$”)”.

### 2.1. Study Selection

**Figure 2:**
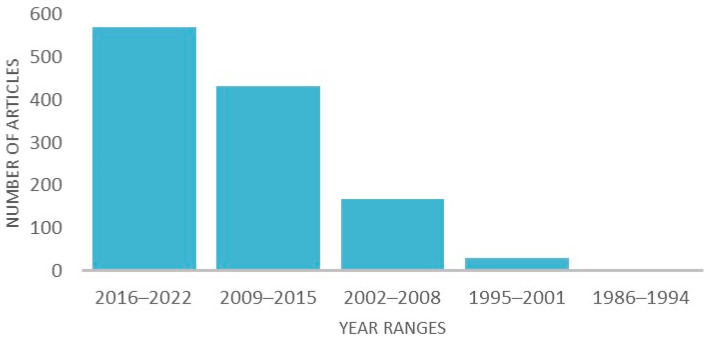
Number of articles found in the systematic search in Web of Science grouped by period of time.

**Figure 3:**
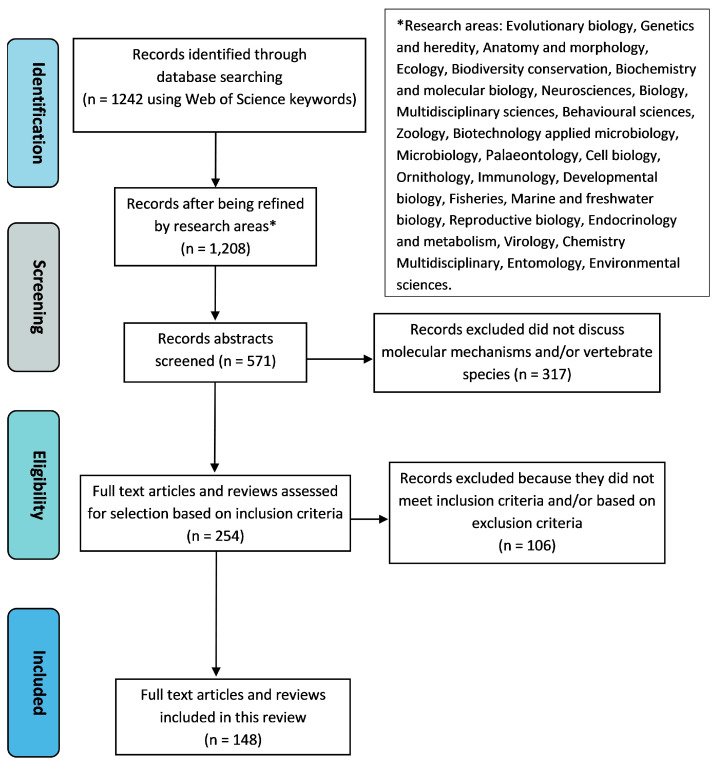
PRISMA flow diagram for systematic review of molecular mechanisms underlying vertebrate adaptive evolution. Records were identified for the years 2016–2022 using the Web of Science database. They were further refined to only include records from relevant research areas. Inclusion criteria: research articles or reviews that investigated or discussed molecular mechanisms involved in adaptive evolution and/or evolution of a trait that was shown to increase fitness. Exclusion criteria: research articles or reviews that described observations suggesting adaptive evolution of a trait (i.e., signatures of selection) but neither offered detailed information about the underlying mechanisms for adaptive evolution nor were supported by other articles that did investigate the underlying mechanism; research articles that deemed their results inconclusive or in need of validation, and/or found no evidence to support the adaptive molecular mechanisms they were investigating; and research articles or reviews that described the evolution of a trait that had not been classified as adaptive. In this search, we selected 148 papers, and 2 additional papers were later included, following the second literature search (see the main text).

The search was refined to only include records from relevant research areas and yielded 1117 records. This was further narrowed down to only cover the years 2016–2022, resulting in 487 records. The search was limited to the last seven years because three previous reviews [1,2,5] discussed similar topics based on the earlier literature. Furthermore, the largest number of relevant papers was published within the covered period (Figure 2). From 571 studies published in 2016–2022, 148 were selected using the inclusion and exclusion criteria (Figure 3).

The additional search focused on reproductive adaptations identified 515 studies, but only 2 studies newly identified in this search met the inclusion criteria. Moreover, 51 studies appeared in both searches, accounting for 9% of papers found in the original search and 10% in the new one. Of these 51 studies, 41 either did not meet the scope of the review, i.e., did not discuss molecular mechanisms in response to environmental factors, and/or did not meet one of the criteria discussed in the methodology. Five studies identified in this additional search are discussed in the section on reproductive adaptations (of which three appeared in both searches and two in the new search only) and the remaining seven studies in other sections.

**Figure 4:**
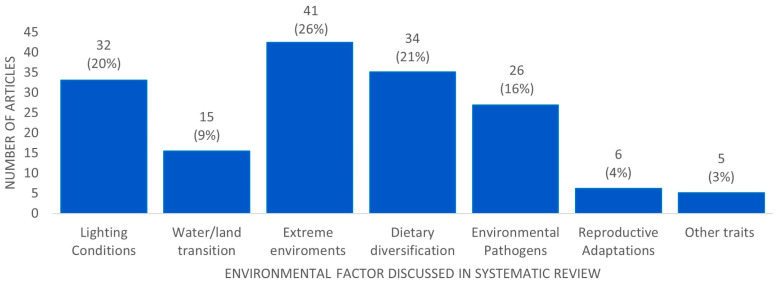
Division of articles by topic, as discussed in this systematic review. The 150 selected articles were grouped into six categories corresponding to the six discussed environmental factors and “other traits” pulled together. A total of 14 articles investigated or reviewed more than one of these environmental factors; consequently, 13 of these articles were discussed in two chapters, and the remaining article was discussed in three.

The addition of two new studies on reproductive adaptations increased the total number of studies included in the systematic review to 150. All these studies are listed in the reference list. The reference list has 152 records, as the first 2 studies cited [1,2] are previous reviews on the topic, which were not identified in the systematic search. The 150 selected studies were grouped into categories corresponding to the six discussed environmental factors and an “others” section for studies that could not be classified into any of these categories (Figure 4). Fourteen studies were assigned to more than one category.

### 2.2. Eligibility Criteria

I. We included research articles or reviews that investigated or discussed molecular mechanisms involved in adaptive evolution and/or the evolution of a trait that was shown to increase fitness.
II. The following articles were excluded:
  i. Research articles or reviews that described observations suggesting the adaptive evolution of a trait (i.e., signatures of selection) but neither offered detailed information about the underlying mechanisms for adaptive evolution nor were supported by other articles that did investigate the underlying mechanism.
  ii. Research articles that deemed their results inconclusive or in need of validation, and/or found no evidence to support the adaptive molecular mechanisms they were investigating.
  iii. Research articles or reviews that described the evolution of a trait that had not been classified as adaptive.

### 2.3. Limitations

Although we aimed to identify as many articles as possible that met the established criteria, it is possible that some relevant articles may be missing from this review if they were not identified based on the set of keywords used in the search and/or were unavailable in the Web of Science database.

## 3. General Aspects of the Molecular Mechanisms Underlying Adaptive Evolution in Vertebrates

Advances in sequencing, proteomics, and bioinformatic technologies enabled researchers to investigate the molecular mechanisms underlying adaptive variation. Sequence variation (e.g., indels (i.e., insertion–deletions) and point mutations) is one of these mechanisms (Figure 1). In some environmental contexts, sequence variation in key conserved sites can result in adaptions, but molecular implications of sequence variation/mutations vary depending on the type of conserved element impacted [6]. Mutations may arise via the insertion of transposable elements, which can result in variations in transcriptional regulation, DNA methylation patterns, and chromosome stability [7]. 

When mutations occur within the coding region, it has the potential to impact amino acid sequences near or within functional protein domains, and/or to affect post-transcriptional regulatory elements such as microRNAs (miRs). MiRs can be tissue-specific, can be developmental-time-specific, and may even be a response to stimuli [8,9,10]. MiR-mediated adaptive evolution can enable a lineage to gain or lose a regulatory sequence rather quickly, modifying regulatory patterns under novel pressure [9]. The modification of regulatory patterns leads to differential transcription. This can also occur due to (1) indirect factors (i.e., mutations leading to functional variation in proteins regulating transcription (e.g., transcription factors)) and (2) mutations in noncoding regulatory regions (e.g., enhancers and silencers) (Figure 1). 

Another regulatory mechanism that can result in differential transcription is epigenetic modifications (Figure 1). Epigenetic changes are molecular modifications that affect genome structure, altering transcription rates by impacting the DNA binding ability of transcription factors and RNA polymerases [11]. Epigenetic modifications have been suggested to be essential for a rapid adaptive response to environmental factors [12]. Epigenetic changes do not always require novel mutations and are reversible (i.e., transient); thus, it can be an effective mechanism for differential gene regulation when organisms are subjected to sudden environmental pressures. Epigenetic variation can provide an immediate response to a sudden environmental change and can be transient. 

Gene losses and losses of function can also give rise to evolutionary novelties, which can be advantageous in some ecological contexts [13]. The idea that gene loss can result in an increase in fitness is a controversial topic. Non-functionality of a gene leads to loss of the protein or proteins coded by this gene, affecting associated phenotypes. Some regulatory elements (e.g., silencers and corepressors) that contribute to gene loss of function have been deemed evolutionary conserved units, implying that gene loss may be adaptive. Exploring the possibility of how gene loss can be advantageous by yielding alternative phenotypes could provide further insight into the complexities of adaptive evolution. 

## 4. Adaptive Evolution in Response to Variation in Lighting Conditions

Visual systems are important sensory systems used for navigation and receiving environmental stimuli. Lighting conditions vary considerably across environments, thus successful colonization of new environments requires adaptation of visual systems. Ecological factors shape visual sensitivities and have an impact on the molecular mechanisms that drive the adaptive evolution of visual structures and/or regulatory mechanisms in vertebrates [6,14,15,16,17]. Adaptation to a novel lighting environment is a complex process that requires changes in lens transmittance and retinal cell structure (i.e., chromatophore ratios and opsin gene expression) [14,15,18,19]. Opsins are photoreceptor proteins that bind to photons, thus interacting directly with an environmental stimulus. 

Adaptive variation affecting retinae structure in response to variation in lighting conditions has been observed across multiple vertebrate species. One of the most common mechanisms is modifications near or within the functional domains of photoreceptors [17,20,21]. A well-described adaptive phenomenon in this regard is spectral tuning. Spectral tuning occurs when sequence variations impact photoreceptor binding sites, modifying the wavelength of the absorbed light that can be detected by photoreceptors [6,22]. 

Mutations in rhodopsin genes (*RH1* and *RH2*) have been observed to cause spectral shifts in fishes, reptiles, and marine mammals as an adaptive response to variation in aquatic lighting conditions or lighting variation associated with living on the forest floor [15,16,22,23,24,25,26]. Spectral tuning associated with mutations in opsin genes (*LWS* and *OPN1LW*) has been proposed as an adaptive mechanism in vision acuity in giraffes [27]. Spectral tuning in the opsins LWS and SWS2 has also been observed as an adaptive mechanism contributing to nocturnal or benthic vertebrates’ ability to navigate low-lighted environments [20,25,28,29]. Both in reptile and fish lineages, relaxed constraints, at times leading to pseudogenization or gene loss, have been observed across species living in dimly lit environments [21,22]. SW1 loss has been proposed to be a beneficial phenotype, contributing to improving opsin sensibility towards longer wavelengths [20]. The shifts in spectral sensitivity are postulated to have occurred due to an increase in crepuscular and/or nocturnal activities of these species. Conversely, spectral tuning in SWS1 was observed in nocturnal reptiles and was proposed as an adaptive mechanism for diurnality [22].

Other molecular mechanisms underlying adaptive variation in visual systems are regulatory changes. In various fish species, the differential expression of enzymes and photoreceptors (e.g., CYP27C1, SWS1, RH2, RHO, and SWS2), and mutations in regulatory regions are suggested to be important molecular mechanisms underlying adaptive visual variation [6,21,25,30,31,32,33,34]. In cichlids, mutations in non-coding regions, specifically miR and transcription binding sites, have been found to be associated with differential transcription of opsin genes [33]. This highlights how a better understanding of where regulatory elements bind in the genome can provide essential information on how differential transcription occurs.

Regulatory modifications resulting in variations in the photoreceptor expression patterns are also suggested as a molecular mechanism underlying visual adaptations to nocturnal/dimly lit environments. In cichlids from dimly lit environments, mutations impacting dimerization properties, subsequently affecting downstream pathways regulated by these receptors, have been observed and are proposed to be adaptive [21]. Regulatory changes have impacted the retinal structure of nocturnal vertebrates to exhibit a higher proportion of rod cells, typically expressing RH1 [16,33,35]. RH1 may be essential for visual adaptations to dimly lit environments [24]. An electric knifefish species from dimly lit environments has been shown to exhibit compensatory substitutions in RH1 thought to be involved in re-establishing the dimerization properties of RH1, compensating for a RH1 mutation associated with the visual defects they exhibit in humans [36].

Mutations in functional domains have also been observed in non-photoreceptor visual systems genes. In the forest-dwelling okapi *LUM*, a gene involved in the phototransduction process exhibits a mutation suggested to impact the protein’s interaction with collagen, subsequently affecting UV transmission in dimly lit environments [27]. Additionally, the functional enhancement of retinae cell surface proteins (GRK1 and SLC24A1) has been proposed as a part of the underlying mechanisms of adaptations of vision in nocturnal birds [37]. 

Adaptive variations in visual systems are complex and can also result in adaptions impacting other aspects of phototransduction processes. In teleost fishes (Teleostei), molecular variations resulting in metabolic changes have been suggested to be involved in these visual system adaptations. A mutation in ATPase VHA involved in the increase in acidification of fish blood cells, leading to an increase in oxygen production and subsequent secretion into the retinae, has been proposed as a key adaptation [38]. This could have contributed to the morphological adaptations within teleost retinas and the adaptive evolution of their visual systems. 

As previously mentioned, nocturnal vertebrates exhibit a higher proportion of rod cells within their retinae. Conversely, relaxed constraints of the visual system loci have been suggested to be involved in the variation and degeneration of nocturnal vertebrates’ visual systems [15,39,40,41,42,43]. Specifically, photoreceptor genes such as *RH1*, *RH2*, *SWS1*, or *SWS2* have been lost under relaxed constraints in some of these species [8,41,43]. However, a unique mechanism of transmutation has been suggested as a means for vertebrates to adapt back to diurnal environments [8,36]. In members of the reptilian *Colubridae* family, which lost retinae cone cells, re-gaining a function has been demonstrated to occur through transmutation, resulting in evolutionary modifications of rods to become cone-like in function [8]. 

Overall, the adaptive variation of vertebrate visual systems is underlined by molecular modifications in retinae. Adapting to varying lighting conditions is a complex process, where many regulatory and structural genes are involved. Research has focused mainly on functional variation within photoreceptors that directly interact with environmental stimuli. However, adaptive variation has also been observed in proteins involved in phototransduction processes and in the expression patterns of photoreceptors. Thus, future studies should also investigate the putative regulatory mechanism, which may impact the expression patterns of photoreceptors as well as the phototransduction process itself.

## 5. Adaptations for Colonization of Aquatic and Terrestrial Environments

Ancestral vertebrates inhabited oceans and, later, transitioned into terrestrial environments. Conversely, some vertebrates regressed toward aquatic environments from terrestrial environments. Multiple molecular mechanisms involved in the vertebrate transition from water to land have been identified [44,45]. Li et al. (2018) investigated the underlaying molecular mechanisms that allowed vertebrates to invade terrestrial habitats by comparing the walking catfish and non-air breathing catfish. Their findings suggested that genes involved in DNA repair, enzyme activation, and small GTPase regulator activity were part of the molecular mechanisms to overcome the increase in DNA damage in terrestrial environments and the variation in metabolic processes. 

The colonization of terrestrial environments also required vertebrates to adapt to hypoxic conditions, terrestrial xenobiotics, and novel environmental stimuli. Gene expansions and tissue-specific regulatory modifications of myoglobin genes (*MB*), sulfotransferase genes (e.g., *SULT6B1*), and olfactory receptor genes (e.g., *ORA1*) have been suggested to be involved in these processes [45]. The tissue-specific differential transcription of genes associated with ion homeostasis, acid–base balance, hemoglobin genes, angiogenesis, elastic fiber formation genes, and mutations in the functional domains of MB are thought to have contributed to overcoming hypoxic conditions in terrestrial environments [45,46]. Adaptations to hypoxic environments are further discussed in a subsequent section of this review.

The transition of vertebrates back to aquatic environments presented a unique evolutionary challenge, as many traits advantageous in these environments had been lost. Interestingly, for some complex traits, the relaxed constraints of specific genes yielded phenotypes that are advantageous in the return to aquatic environments. Specifically, the miR-based downregulation of genes has been suggested to be involved in adaptive variation for diving and overcoming hypoxic conditions. Tissue-specific variation in the microRNome of the deep diving Weddell seals suggests an important role of post-transcriptional regulatory mechanisms in marine mammal adaption to the aquatic environment. The tissue-specific differential expression of miRs targeting genes associated with hypoxia tolerance, anti-apoptotic pathways, and nitric oxide signal transduction was observed in Weddell seals [10]. MiR-mediated post-transcriptional regulation has been shown to be involved in downregulation, a mechanism that results in a substantial decrease in the amount of protein translated, which mimics the gene being “turned down” or “off” (Figure 1A) [9]. This suggests that the functional loss of some genes could potentially yield advantageous traits in marine mammal diving adaptions [10]. 

Eukaryotic regulatory pathways are complex, and losing a protein in the pathway which modifies it could potentially yield an advantageous phenotype by modifying instead of losing the trait [47]. This is the case for matrix metalloproteinase (*MMP12*), epidermal and hair development genes (*DSC1*, *DSG4*, *TGM5*, *GSDMA*, *LYG1*, and *LYG2*), and keratin genes (*KRT9* and *KRT20*). MMP12 is involved in extracellular matrix breakdown, and its loss impacts pulmonary elasticity in a way that allows marine mammals to renew ~90% of their air in a single breath, an advantageous trait for diving [47]. The loss of the aforementioned epidermal and hair developmental genes resulted in modifications of the respective developmental pathways, yielding alternative traits of a thicker epidermis and hair loss traits in cetaceans and sirenians, suggested to be advantageous for the aquatic environments [47,48,49]. 

Loss of other functional proteins has also been suggested to have yielded advantageous traits in marine vertebrates. Relaxed constraints and subsequent gene loss of some olfactory and taste receptors genes (e.g., *GNAT3* and *CALHM1*) observed in marine birds and mammals have been suggested to be advantageous traits by masking the smell and taste imbued onto their prey by the seawater [50,51]. Another example is the relaxed constraint of erythrocyte specific enzyme (AMPD3), which causes a reduced affinity to oxygen and a threefold increase in ATP in erythrocytes, an advantageous trait for diving [47]. 

Mutations in proteins with regulatory functions in cellular pathways (*ACAN*, a growth inhibitor), osteogenesis, and gene regulation (*PIT-1*, *HOXD11*, *HOXD12*, *HOXD13* and *MLL*) have been proposed to be molecular mechanisms involved in the adaptive variation of the cetacean body architecture. The lineage-specific mutations are predicted to impact the structure and functionality of these proteins [52]. As in other vertebrates, they result in a body size reduction as well as shortened limbs and trunk [53]. Mutations in the aforementioned transcriptional factors impact their regulatory functions (e.g., binding affinity to target genes, chromatin remodeling, and histone modification), consequently modifying the transcriptional patterns of their target genes [11,54]. Target genes include genes involved in determining vertebrate body size (e.g., *ELK1*, *LHX3*, and *PITX1*), suggesting that these mutations are involved in the adaptive variation of cetacean body size and skeletal morphology [11,53,55].

The functional variation and expansion of genes involved in multiple cellular functions have also been proposed as underlying molecular mechanisms involved in adaptions to aquatic environments. Mutations in cetacean ATPase (*ATP8*) have been suggested to result in metabolic adjustments beneficial in their marine environments [56]. Mutations in a reproductive gene (*FSHR*) are suggested to be involved in adaptations of marine mammals’ reproductive systems [48]. Finally, the expansions of genes involved in multiple cellular processes (i.e., cellular response, oxidative stress, oxidation reduction, and hydrogen peroxide response) have been identified in cetaceans, other aquatic mammals, and semi aquatic mammals. They are likely involved in adaptations to hypoxic conditions, aquatic pathogens, novel energetic metabolic demands and nervous system adaptations [48,57,58,59]. 

Transitions to terrestrial and aquatic environments are complex evolutionary processes that require variation associated with both environmental stimuli (e.g., xenobiotics) and navigating these novel environments (e.g., diving). Due to the complex regulatory dynamics of eukaryotic gene expression, modification or even loss of regulatory proteins have yielded phenotypes that are advantageous in specific environmental contexts. Thus, future research in this topic would benefit from approaches that investigate the role of specific biochemical pathways involved in relevant cellular functions and gene regulation.

## 6. Adapting to Extreme Environmental Conditions

Extreme environments can occur due to natural ecological factors as well as anthropogenic factors (e.g., pollution). Pollution can cause an array of environmental stressors for organisms. Amazonian teleost fishes living in polluted environments have been observed to exhibit an increase in transposable elements, which are thought to contribute to their ability to endure these stressful conditions [7]. In snow finches from the Tibetan Plateau, mutations in a DNA repair gene (*DTL*) have been suggested to be an adaption for their high-altitude environments [59]. Furthermore, mutations in metabolic enzymes (*GST*) are thought to be involved in modulating specificity or potency of the detoxification and metabolic activation of xenobiotic pathways in mammals [60]. 

### 6.1. Hypoxic Environments

Hypoxic conditions (i.e., low oxygen) exist both in terrestrial and aquatic environments and can negatively impact biological processes. Functional mutations and regulatory modifications are suggested to be molecular mechanisms underlying vertebrate adaptations to hypoxic conditions [61,62]. Mutations improving binding affinity in the oxygen-transporting molecule (HB) have been identified in multiple vertebrate lineages [5,46,63]. The convergent evolution of a hypoxia inducible factor (*EPAS1*) has been observed in multiple unrelated vertebrate species living in hypoxic conditions in Tibet [64]. This suggests that functional mutations in proteins that either directly interact with oxygen molecules or are involved in the modification of cellular oxygenation, have been essential for adapting to hypoxic environments. Physiological adaptations within respiratory systems can also be essential in hypoxic environments. Variations in regulatory pathways have also been proposed as a mechanism underlying lung adaptations in yaks living in hypoxic environments [62].

In Tibetan fish species living in hypoxic environments, rapid evolution was inferred for genes enriched in hypoxia and energy metabolism [61,65]. The co-evolution of genes that enhance the function of the hypoxia-inducible factor was also suggested to be involved in adaption to these conditions in fishes [61]. Mutations inhibiting target protein-binding affinity in a regulatory protein (VHL), whose function is targeting the hypoxia-inducible factor to degradation, has been observed in tree unrelated fish lineages living in hypoxic conditions [61]. Mutations resulting in the functional variation of proteins involved in metabolic processes and/or pathways have also been suggested to be involved in mammalian adaptations to hypoxic environments. Mutations in genes involved in metabolic processes (e.g., rate limiting enzymes within the gluconeogenesis pathway (LDHA, LDHD, PC, PCK1, FBP1, and GPI), anaerobic respiration (ALDOA and ENO), oxidative phosphorylation proteins (NDUFA9, NDUFA10, NDUFAB1, NDUFC2, and NDUFV3), the hypoxia inducible pathway, and the mitochondrial NADH dehydrogenase complex have been suggested to be involved in mammalian adaptations to hypoxic environments [63,66,67].

Modifications in transcriptional patterns have also been proposed as molecular mechanisms involved in adaptive variation to hypoxic environments. For instance, both tissue-specific differential expression and mutations of cytokine erythropoietin (*EPO*) enabled schizothoracine fish to adapt to their hypoxic environment [68,69]. Some of the mutations identified in EPO have been shown to confer antioxidative and antiapoptotic properties to the protein, both of which are beneficial in hypoxic environments [69]. Finally, developmental and tissue-specific differential expression of miRs enriched for genes involved in multiple cardiac processes (e.g., ischemic postconditioning, perfusion, and angiogenesis) have been associated with managing challenges associated with hypoxic conditions in Weddell seals [10].

### 6.2. Extreme Aquatic Environments

An environmental factor for which aquatic vertebrates had to adapt is the salinity content of the water (i.e., freshwater vs. saltwater). For most saltwater species, freshwater is an extreme environment and vice versa. Transitioning from one to the other requires specific adaptations. The differential expression of genes associated with metabolic processes (e.g., homoeostasis, transmembrane transport, ion-exchange, osmoregulation, blood traits, and tooth morphology) has been observed between freshwater and saltwater stickleback ecotypes [70,71,72,73]. Additionally, in sticklebacks, it has been proposed that some of the marine-environment-specific advantageous variation can be inherited in clusters as a unit [74]. The differential expression of genes including transcription factors associated with stress response and immune responses has been observed in the tongue sole fish in response to a variation in the salinity content of the water [75]. Both cis- and trans-regulatory variations are suggested to modify the expression patterns of the genes (e.g., *GLA* and *IGFLR1*) associated with these processes [70,71,73]. The environment-specific adaptive variation in both sticklebacks and cod fishes has been suggested to be mediated by chromosomal rearrangements [76]. In reptiles and amphibians, gene expression variation mediated by transposon activity has been proposed as one of the mechanisms for adaptations to marine environments [77]. 

Regulatory variation is another molecular mechanism involved in adaptive morphological variation to freshwater environments. Mutations in cis-acting regulatory elements (i.e., transposon insertion and/or deletions) of genes associated with morphological and skeletal development (i.e., *PITX1*, *EDA*, *KITLG*, *BMP6,* and *GDF6*) and nutrient intake (i.e., *CSAD* and *CTH*) are thought to be involved in the emergence of adaptations for freshwater environments in sticklebacks [72,78,79,80,81]. Regulatory variation in one of these genes (*PITX1*), which underwent deletion of an enhancer region, is suggested to have occurred due to increased mutation rates resulting from the DNA breakage propensity of said region [82]. Similarly, the deletion of an enhancer of *EDA* is thought to be pleiotropically involved in variation in multiple traits associated with morphological and feeding adaptions to freshwater environments (i.e., lateral plate count, number, and patterning of posterior lateral line neuromasts) [81,83]. 

Mutations resulting in the functional variation of *SLC12A3* and the truncation of *MSX2* have also been suggested to be involved in adaptations to freshwater environments. Mutations observed in *SLC12A3* in Atlantic and Baltic herring have been proposed as necessary adaptive variations for osmotic balance in response to environmental differences in salinity [15]. Mutations in *MSX2* resulting in alternative splicing, which in turn results in higher expression of a truncated isomorph, are thought to be involved in the adaptive reduction in spine length in freshwater sticklebacks [84]. Finally, mutations in choriolytic enzyme loci impacting hatching in herring have been suggested to be involved in reproductive adaptive variation to salinity content in aquatic environments [15]. 

Another environmental challenge in aquatic environments is highly acidic or highly alkaline waters. Mutations in *VIPR1* and mitochondrial proteins (e.g., COX3 and COX1) of Amur ide fish and *Poeciliidae* fish have been associated with adaptations to environments with high sulfuric acid concentrations and alkaline environments, respectively [85,86,87]. The duplication of *NPR1*, *ZP*, and *VMO1* genes have also been proposed as mechanisms involved in adaptations to alkaline environments. The expansion of these genes yielded an adaptive variation of osmoregulatory mechanisms and a thickening of the egg chorion, respectively [88]. 

The differential expression of *NPR1*, *CA*, *GST*, and *SOD* genes is suggested to underlie homeostatic adaptive variation to alkaline environments in teleost fish [87]. The differential expression of metabolic genes (e.g., *PKLR*; *FBP2*, *G6PCB*, and *PCXB*) and genes associated with DNA replication and repair, and proteasome assembly have been suggested to be involved in compensatory growth [89]. Compensatory growth, i.e., rapid growth occurring after adverse environmental conditions have dissipated, is an advantageous trait in transient extreme environmental conditions.

### 6.3. Temperature Extremes

Extreme high or low temperatures are another environmental factor that can create adverse conditions. These extremes can lead to water loss, metabolic changes, and/or cell viability. Adaptations in functional domains of proteins (*ABCA12*) and differential expression (*ATP1A1*) have both been proposed as molecular mechanisms underlying adaptations to high temperatures [90,91]. In birds and mammals, mutations in a temperature-sensitive receptor TRPM8 have been suggested to be involved in cold-tolerance adaptations [41]. The combined effect of mutations in *PGC-1*, which regulate the expression of mutated variants of thermogenin (*UCP1*), affected the heat-generating capacity of brown adipose tissue, enabling mammals to adapt to low-temperature environments [63]. 

Expansions of the 118 gene families involved in metabolic processes have been suggested to be involved in adaptation to low-temperature environments [15]. Epigenetic modifications (e.g., DNA methylation) are thought to be involved in the adaptation to transient low temperatures in winter skates [92]. De novo gene birth has been identified as the mechanism by which similar anti-freeze proteins (AFGP) arose in two unrelated teleost fish species [93]. The rare event of de novo gene formation occurred in both species: duplication of noncoding DNA elements, which over time gained the functional elements to become a gene (i.e., TATA box, open reading frame, and a promoter). 

## 7. Adaptations to Dietary Changes

### 7.1. Metabolic Adaptations

Access to food/nutrients is an important ecological factor that often influences evolutionary adaptations. The mechanisms underlying food selection can be complex, requiring adaptations in digestive systems and/or feeding apparatus. Teleost fishes’ adaptive variation within insulin-based metabolic processes arose via a combination of gene duplications, pseudogenization, and retrotransposition [94]. In the Gekkonidae lineage, functional variation in IGF-1 resulting in a switch between metabolic pathways is suggested to be involved in their dietary diversification [95]. The adaptive evolution of mammalian metabolic systems has been found to have occurred mainly due to mutations leading to structural and functional variations in proteins [4]. 

Adaptations for diet diversification in birds resulted from the combined effect of gene duplications, losses, and functional protein variation in digestive enzymes [96]. Mutations in a carbohydrase (*AMY*) and a lipase (*CYP7A1*) were commonly observed amongst specific bird groups (i.e., carbohydrase in seed eaters and lipase in meat eaters) suggesting an association with diet diversification [97]. In mammals, duplication and functional variation in an immune system gene, *RNASE1*, and a G-Protein-Coupled-Receptor have been proposed as mechanisms underlying adaptations toward herbivory [97,98]. 

Cetaceans experienced a dietary shift from herbivory to carnivory. Mutations in functional domains of multiple proteins (PGA, LIPF, ACAD9, EHHADH, and PLRP2) have been proposed to be associated with this shift [54,99]. The functional implications of these mutations have not been described for all these proteins. However, the mutation in PGA increases binding to the ligand, while the one in PLRP2 is thought to be involved in enabling calves to digest fattier milk. Another mammal that experienced a dietary shift was the domestic dog, who adapted to feed on human waste. This transition could have been facilitated by an emergence of novel retrogenes involved in lutathione biosynthetic/metabolic processes and toxic substance response [100]. 

### 7.2. Non-Metabolic Adaptations

In giant pandas, the pseudogenization of bitter taste receptors (TAS2R) was suggested to have facilitated their shift to an almost exclusive bamboo diet. Bamboo has bitter-tasting substances as a defense mechanism, and the loss of bitter taste receptors is thought to have enabled giant pandas to overcome this challenge [101]. Additionally, epigenetic modifications increasing the expression of thiosulfate sulfurtransferase have also been proposed as an adaptation in red and giant pandas to overcome the toxicity of bamboo [102]. Polar bears underwent a dietary shift to a hypercarnivorous diet, which is thought to have resulted in losses in the copy number of genes associated with a digestive system, (e.g., *AMY1B* and *NOX4*) and olfactory receptor genes [103]. These copy number losses are proposed to have yielded advantageous traits, enabling polar bears to increase fat storage (*NOX4*) and to develop a more acute olfactory system [103].

Dietary shifts can inadvertently cause imbalances in nutrient availability or toxicity issues. Vampire bats exhibit a diet exclusively based on blood and have a unique evolutionary strategy to overcome associated nutrient deficiencies. Vampire bats have a mutational bias favoring cytosine and not favoring thymine, which subsequently increases threonine in their proteomes, enabling them to overcome inherent amino acid limitations in their diet [104]. Convergent epigenetic changes in both red and giant pandas have been proposed as the molecular mechanism behind the differential expression patterns of genes involved in lipid digestion (*DGAT2*, *SLC2A3*, and *PARL*), enabling them to overcome nutritional challenges of their low-lipid diet [102].

In 31 bird species, mutations in alanine-glyoxylate aminotransferase (AGT) modifying protein localization have been suggested to be involved in enabling them to overcome carnivory-associated toxicity issues [105]. Similarly, both in colubrid snakes and mammals, mutations in voltage-gated sodium channels pores (*SCN4A*, *SCN8A*, and *SCN9A*) have immunized some species to tetrodotoxin (i.e., toxic compound secreted by some amphibians (e.g., *Taricha* newts) as a defense mechanism against predators [8,106,107]. Amphibians that secrete tetrodotoxin also have mutations in voltage-gated sodium channels, conferring them resistance to the toxin [108]. These mutations are suggested to have been facilitated by duplication events.

### 7.3. Anatomical and Behavioural Adaptations

Dietary shifts can also require the emergence of novel traits to catch and/or consume novel food items. Snakes have developed venom-delivery systems to subdue their prey. The molecular mechanisms underlying this adaptation are complex and vary amongst lineages. The current consensus is that a combination of neofunctionalized genes and tissue-specific differential transcription is the main mechanism underlying the emergence of this trait [8]. Some birds have evolved tactile foraging behaviors to catch their prey. An increase in expression mechanoreceptors associated with tactile sensitivity has been observed in three species of birds exhibiting this behavior [109]. 

Dietary shifts often require changes in craniofacial structures, and regulatory modifications have been proposed to play a part in such changes. In sticklebacks and cichlids, an adaptive variation in craniofacial development, including teeth number and morphology, is suggested to occur due to differential expression patterns of developmental genes (*ODAM, UNK*, *SCPP5*, and *RPFA*), including cis-regulatory (*TFAP2A* and *BMP6*) and/or trans-regulatory changes [72,110,111,112]. Adaptive variation in mammalian teeth shape has also been associated with mutations resulting in functional variation in developmental (*BARX1*, *EVE1*, *LHX7*, *LHX8*, and seven *HOX* genes) and regulatory (*BMP2*, *BMP4*, *DLX2*, *EDA*, *EDAR*, *PAX9*, *PITX2*, *RUNX2*, and *SHH*) genes [113,114]. In sea horses, African cichlids, and some mammalian species, the loss of conserved elements and various morphological genes (e.g., *TBX4*, *SALL1A*, *SHOX*, and *IRX5A*), and a negative regulation leading to effective loss of genes affecting morphological features (BMP) have been associated with adaptive phenotypes [13,114,115]. Loss of specific genes and regulatory elements contributed to morphological changes in the head (e.g., teeth and jawbone), resulting in advantageous evolutionary feeding traits/novelties [13,114,115]. 

Behavioral adaptations are involved in dietary shifts and often require complex variation involving multiple genes. Differential expression of the developmental gene *EDAR* is thought to impact their predatory behavior in teleost fish. Lower levels of *EDAR* expression have been associated with an increase in predatory behavior in both zebrafishes and Mandarin fishes [116]. Conversely, in cave fish species, mutations in MAO and OCA2 are suggested to be involved in adaptive behavioral changes (e.g., reducing predatory, schooling, or sleeping behaviors) [43,117]. In cetaceans pseudogenization (*MCPH1*) and mutations in neurodevelopment genes (*WDR62*, *CDK5RAP2*, *CEP152*, and *ASPM*) are thought be involved in an incrementation in brain size [118]. These developmental and anatomical modifications can modify or add complexity to feeding behaviors. 

## 8. Environmental Pathogens

Vertebrate immune systems are characterized by adaptiveness under the co-evolutionary arms race with pathogens. Sticklebacks display habitat specific (i.e., lake vs. river) differential expression of immune system genes; thus, gene regulation underlies adaptations in the presence of habitat-specific pathogens [119]. Amino acid changes near or within the functional domains of immune system proteins are one of the main molecular mechanisms underlying adaptations to environmental pathogens [4,120]. Mutations giving rise to habitat-specific functional adaptive variation in immune system genes (*IGHM*, *IGHE IGH*, *CD14*, *CD40*, *CD80*, *IFNAR2*, *LY96*, *TAB 1*, *TICAM1*, *TLR4*, *MDA5*, chicken type lysozyme, and *pIGR*) have been observed in different vertebrates and can happen through insertions (e.g., MUC7 in primates) or gene duplications [121,122,123,124,125,126,127,128]. For the pIGR specifically, research suggests that the observed mutations occurred through an insertion of transposable elements [127].

Functional variation has also been observed in non-immune system proteins. In several species of Galliformes birds, a single amino acid change in MX Dynamin Like GTPase, confers resistance to avian influenza [129]. In gaur, an Asian bovid, mutations causing a structural conversion in the mRNA-editing protein APOBEC3Z3 enabled the protein to acquire resistance to degradation by the infectivity factor of the Jembrana disease virus [130]. 

Gene duplications and the neofunctionalization of genes have occurred in multiple vertebrate immune system gene families (e.g., *MHC*, *NLPRP3*, *CD22*, immunoglobins, *TLR*s, and *SSC4D*) [126,131,132]. Among the most studied gene families are immune pattern recognition receptors, and *TLR* gene family is of particular interest due to their involvement in pathogen detection. Environmentally specific duplication and/or neofunctionalization of TLR genes have been observed across fish, reptile, and bird species [133,134,135,136,137]. Environmentally specific functional variation in pattern receptors including TLR family genes, impacting binding affinities, and/or imparting resistance to environmentally specific pathogens has been observed in mammals, birds, and reptiles [123,134,137,138,139,140,141]. The emergence of novel pathogen resistances enabled vertebrates to colonize novel environments [141].

Within the *Rhinolophoidae* and *Pteropodidae* bat lineages, indels have been suggested to lead to functional variation, impacting the binding affinity of TLR8, thus improving pathogen recognition [142]. Gene losses are also thought to be essential in the adaptive evolution of vertebrate’s immune systems. Negative selection leading to the loss of genes’ *NF-kB*, targets of TLRs, has led to conservancy of functional domains and copy numbers from fish to mammalian vertebrates [143]. Gene expansions, gene losses, and mutations leading to neogenization and/or functional variation, particularly impacting binding affinities and protein–protein interactions, are the main mechanisms known to produce immune systems adaptive variation.

## 9. Reproductive Adaptations

Reproductive adaptations have also been necessary for the colonization of some environments. In bony fishes, the duplication of zona pellucida genes (*ZP*) has been proposed to play a role in reproduction via external fertilization and by successfully hatching eggs, providing an additional layer of protection under the constraints of the environmental factors [144,145]. Multiple gene-duplication mechanisms have been identified in this process (i.e., genome duplication, gene block duplication, and tandem duplication). Conversely, vertebrates with internal fertilization exhibit signs of relaxed purifying selection, pseudogenization, and subsequent reduction in number of *ZP* genes [144].

In platyfish, the duplication of *C6AST* genes has been associated with the adaptive evolution of internal fertilization and ovoviviparity [13]. In seahorses, the duplication of *C6AST* arose independently, and it is involved in the evolution of male pregnancy in these fish [13]. The species also exhibits duplication and neofunctionalization of the *PASTN* genes, which are thought to be involved in the adaptive evolution of internal fertilization [13]. These molecular mechanisms (i.e., gene duplications and neofunctionalization) are also involved in mammalian reproductive adaptations. *CRISP* genes are involved in sperm adaptions to increase male reproductive success [146]. 

The evolution of novel reproductive strategies in response to environmental factors can require adaptations in non-reproductive structures. Some species of anglerfish have evolved a unique reproductive strategy to overcome challenges in their deep-sea environments: parasitic males. Males in these species have evolved to be parasitic and are permanently attached to the females. Without the necessary immune system adaptations, the parasitism of the male would illicit an immune response in the female, which could potentially kill the males. These adaptations involved mutations impacting the function of immune system proteins (e.g., substrate binding affinity), differential regulation (e.g., reduced expression), and gene losses (e.g., pseudogenization) (*MHC* and *CD* gene families) [147]. 

Another reproductive strategy that required adaptation of non-reproductive structures is acoustic communication used to find and/or compete for mates. Concave-eared frogs live in environments with background noise that can obscure their acoustic communication. Supersonic hearing has been proposed as an adaption phenotype to background noises in their environments. Differential transcription in genes involved in neurogenesis and sensory perception has been proposed as one of the mechanisms underlying the evolution of this trait [148]. Multiple genes observed to be differentially transcribed in male and female anglerfish are involved in gene regulation or have roles within cellular pathways [148]. Thus, gene duplication, neofunctionalization, differential transcription, and gene losses have been important molecular mechanisms in reproductive adaptations.

## 10. Other Environmental Factors

### 10.1. Changes in Circadian Rhythm

Another environmental factor that vertebrates contend with is day/night cycles. In Artic reindeer, mutations in a clock gene (*PER2*) resulting in loss in binding affinity to another circadian rhythm protein (CRY1) are suggested to impact their sleep/wake cycles, enabling them to adapt to unusually long nights in their environments [3].

### 10.2. Morphological Adaptations

The colonization of novel habitats often requires morphological adaptations. Indels in EDA have been associated with the advantageous loss of scales in underground snow trout inhabiting the Tibetan plateau [149]. Mutations in the transcription activation domain of mammalian MLL, a methyltransferase involved in epigenetic regulation by chromatin remodeling of *HOX* genes, imply modifications in *HOX* expression [11]. *HOX* genes play a key role in morphological development, and differential transcription of these genes due to MLL mutations is one of the mechanisms underlying adaptive morphological variation in mammals [11]. The unique morphological features in seahorses have been suggested to have emerged due to the loss of noncoding regulatory elements in genes associated with vertebrate morphology (e.g., regulation of transcription, pectoral fin morphogenesis, steroid hormone receptor activity, regulation of the fibroblast growth factor receptor signaling pathway, and O-acetyltransferase activity) [13]. Epigenetic regulation, methylation, impacting gene expression, and subsequent regulatory pathways have also been proposed as a mechanism underlying adaptive physiological changes in muscle properties of pelagic migratory fishes [150].

## 11. Limitations of the Study

A systematic review approach has many advantages, including clearly defined selection criteria and reproducible results. This approach also eliminates conscious or unconscious bias in the article selection, which can occur in the case of a traditional review, where the list of articles cited depends on the choice made by the authors. However, systematic reviews also have some inherent limitations, as they can be subject to errors associated with the search algorithm as well as human error. In this instance, the literature review was limited to the results of a search with a specific query and a specific search engine. Consequently, if publications within the scope of this review were unavailable in Web of Science and/or did not have the keywords in the query within their titles, keywords section, and/or abstracts, they were not included. This could have led to the seeming underrepresentation of some topics (e.g., reproductive adaptations). Additionally, there is an apparent overrepresentation of findings based on the use of model organisms. This is unlikely to result from any bias in the choice of keywords but instead reflects the fact that studies on model organisms tend to be at the forefront of the progress in any biological discipline and are overrepresented in the literature in general. Finally, the review cites multiple papers by the same authors or research groups. Given that the studies were identified using objective criteria, this outcome does not result from any preferences of the review authors but reflects productivity of particular researchers or groups working in the field discussed in the review.

## 12. Future Research

There are multiple molecular mechanisms underlying adaptive evolution in vertebrates. For the 157 genes presented in this review, 4 major molecular mechanisms underlying adaptive evolution were described: sequence variation/mutation (56%), gene expansions (17%), differential transcription (12%), and gene loss or pseudogenization (12%) (Table 2). The most frequent molecular mechanism of adaptive variation is mutation, either in conserved coding or non-coding regions [2]. Mutation mainly impacts eukaryotic transcriptional regulatory mechanisms (e.g., enhancers and miRs) or functional domains of proteins (e.g., ligand binding and dimerization) [4,32,33,120]. Interestingly, positively selected sites tend to occur close to each other within the proteins, resulting in functionally important regions [4]. Thus, it is important to analyze the location of such regions within the genome, which could provide further insight into the molecular mechanisms associated with adaptive variation. For instance, DNA fragility has been proposed as the reason for increased mutation rates within specific regions of the genome [82]. 

Mutations within non-coding regions can result in the emergence of novel regulatory elements (e.g., miRs). Duplication is one of the main molecular mechanisms by which novel miRs arise and undergo neofunctionalization in vertebrates [9]. Mutations within regulatory elements can affect gene expression, and these modifications in gene expression can yield adaptive variation [32]. Mutations that modify translational and post-translational processes can also yield adaptive variation. Novel ubiquitination sites can impact both protein localization and protein structure. As vertebrate lineages diverged, novel ubiquitination sites emerged in proteins associated with multiple cell processes (e.g., differentiation and motility), suggesting that this was a novel adaptive variation [151]. To gain a better understanding of the mechanisms underlying vertebrate adaptive evolution, a deeper look into regulatory modifications in both genetic and cellular pathways is needed. The gene loss of a function should also be considered, since the loss of regulatory elements in cellular pathways can yield advantageous phenotypes [81,82].

Adaptive variation can occur in specific regions and even more so in clusters, meaning that larger functionally important regions instead of specific sites need to be conserved [74]. There is a growing body of research proposing that chromosomal rearrangements and inversions are involved in maintaining adaptive genotypes [76]. This could also be possible in some immune system proteins and xenobiotic-metabolic enzymes, as these proteins tend to have evolutionary conserved functional domains [4]. In teolosts, RNA interference specifically of piwi-interacting RNA (piRNA) has been suggested to be involved in regulating the insertion of transposable elements [152]. PiRNA are a class of noncoding RNA molecules involved in maintaining germline integrity through transposon silencing, and consequent transcriptional and post-transcriptional regulation [152]. This mechanism has been shown to be a key element in ensuring genome integrity, which includes genomic regions with adaptive variation [152]. Thus, RNA interference could be an important molecular mechanism affecting the conservation of adaptive genomic regions. 

Epigenetic regulatory mechanisms present a promising area of study for understanding rapid adaptive evolution [5]. There are multiple examples of adaptive variation due to differential transcription [32,68,112,145]. However, there is a gap in our knowledge of how epigenetic variation arises. Transposon activity has also been proposed as a mechanism involved in adaptive variation within regulatory mechanisms [77,127]. Altogether, these developments suggest that there could be mechanisms in place that leads to an increased occurrence of adaptive variation within specific regions of the genome. Investigating the genome structure could provide further insights into how adaptive differential transcription occurs, particularly transient adaptive variation, which is likely regulated by epigenetic regulatory mechanisms. 

Investigations into the mechanisms of adaptive evolution have “evolved” with novel technologies and the continued increase in accessibility to genomic, transcriptomic, and proteomic data. Now, it is possible to go beyond identifying signatures of selection and to investigate the precise molecular mechanisms conferring adaptive variation under specific environmental contexts for particular traits. The key role of regulatory mechanisms in adaptive evolution has become evident. Thus, going forward, it may be worthwhile to continue employing approaches that investigate not only mutations and differential transcription but also how these changes have been conserved, and to consider the potential beneficial impact of losses (e.g., downregulation or loss of function). Further research into regulatory elements (e.g., silencers, enhancers, miRs, piwiRNAs, and epigenetic modifications) could provide significant insight into the mechanisms by which vertebrates permanently or transiently adapt to their environments. 

## 13. Conclusions

Based on the research thus far, the main molecular mechanisms underlying adaptive evolution in vertebrates are sequence variation/mutations. These mutations commonly occur in regulatory elements or functional domains of proteins, leading to regulatory modifications such as changes in gene expression, or cellular and/or signaling pathways. Therefore, modifications in gene expression patterns, and cellular and/or signaling pathways are important molecular mechanisms underlying adaptive variation identified in this review. Thus far, most studies on this topic have aimed to identify selected regions and mutations associated with adaptive phenotypes, although there has been an increase in studies investigating differential transcription. Studies on the molecular impact of mutations (e.g., the effect on the translated protein or what regulatory changes led to the differential transcription) have been rare. Only one of the reviewed studies employed both a genomic analysis and functional assays to investigate the impact of the mutation they identified [117]. The more frequent use of such an approach is needed to advance our knowledge of molecular mechanisms of adaptive evolution. More research into RNA mediated regulation (e.g., miRs and piwiRNAs) and epigenetics as pertaining to adaptive evolution is also needed. Currently, such studies may be limited by the accessibility to samples, the high costs of the necessary assays, and/or access to fully annotated genome sequences. As the field continues to grow, these limitations should continue to be reduced.

Cellular regulatory pathways are complex, and changes in function (e.g., binding affinity) or absence of a protein can considerably impact phenotypes. The possibility that gene losses could be adaptive is a debated topic and is subsequently also understudied [13]. There is evidence that miRs’ sequences can be conserved elements and that they have only been reported to downregulate genes, which may have a similar effect as gene loss. There are also other mechanisms of gene downregulation [9,10]. Some of them can be permanent modifications (e.g., mutation in a regulatory element) or transient (e.g., epigenetic). In some contexts, the downregulation or loss of a gene may yield an advantageous phenotype, e.g., the downregulation or loss of a gene in a regulatory pathway may result in a downstream effect that changes the final molecular response and yields a novel phenotype. Finally, the genome architecture should also be further explored, as there have been some interesting biochemical findings explaining why mutations are more likely to occur in some areas over others.

## Figures and Tables

**Table 1:** Brief descriptions of the molecular mechanisms illustrated in Figure 1.

Concept	Brief Description
Point mutation	Change in DNA sequence by a single nucleotide; it can be an insertion, duplication, or deletion.
Insertion	DNA sequence variation by insertion of one or more nucleotides.
Duplication	Modification in DNA sequence by duplication of a stretch of nucleotides (i.e., one or more).
Deletion	Change in DNA sequence by excision of one or more nucleotides.
Upstream regulatory regions	Regions upstream (before) from the core promoter to which transcription factors (TF) or coactivators can cis-regulate. Transcription factors can activate or increase transcription when bound to enhancers, while repressors can decrease transcription when bound to silencers.
Core promoter	Regulatory region where the transcription initiation complex (TIC) binds, as it has the transcription start site and can include a thymidine adenine-rich region (i.e., TATA box) where the TIC is recruited.
Coding region	DNA sequence that codes for the pre-mRNA, which is later modified to the mRNA and translated into proteins.
Upregulation	Regulatory change that leads to an increase in transcription of specific mRNA.
Gene duplication	Refers to the duplication of a complete gene.
Neogenization	DNA sequence gains a novel gene function and can occur in previously non-coding regions, more commonly in duplicated genes.
Pseudogenization	Process by which a gene either loses a function or becomes completely non-functional.
Downregulation	Regulatory change which leads to a decrease in transcription of specific messenger RNA (mRNA).
microRNAs (miRs)	Small RNA fragments that can hybridize with target mRNA and can lead to its decay, thus downregulating it.
Epigenetic modifications	Molecular modifications (e.g., acetylation, methylation, and phosphorylation) to histones, proteins involved in the packing of DNA. These modifications can impact chromatin condensation and/or RNA polymerase activity.
Chromatin	A protein–DNA molecular complex that is the natural state of Eukaryotic genetic material.
Transcriptional regulation	Processes that impact the rate of transcription, either upregulating or downregulating; these can be cis-regulatory elements (e.g., enhancers or silencers) or components of the transcription initiation complex (TIC) (e.g., co-activators and co-repressors).
Enhancer	Upstream regulatory element that can cis-regulate transcription (upregulation) by interacting with activator transcription factors, which in turn can interact with co-activators in the TIC.
Silencer	Upstream regulatory element that can cis-regulate transcription by interacting with repressor transcription factors, which in turn can interact with a co-repressor in the TIC, for disassociation from DNA.
Post-transcriptional regulation	Regulatory mechanisms that operate upon the mRNA transcript itself or in its maturation process.
microRNA-mediated mRNA decay	A mechanism in which mi-R hybridize with target DNA and recruit the Argo complex, which leads to removal of the protective elements of the mRNA: its cap and polyadenylation tail, which leads to degradation and subsequent downregulation of said transcript.
Splicing	A process that occurs during mRNA maturation, where segments of the raw transcript (pre-mRNA) are removed.
Alternative splicing	Alternative mRNA maturation processes that result in variations in the final mature RNA transcript and subsequent variation in the translated amino acid sequence, which in turn can result in functional variation in protein properties (e.g., dimerization, folding, and ligand affinity).

**Table 2:** Complete list of genes/proteins names discussed in this review, their ontology, and their mechanisms of evolution. D: differential transcription; S: sequence variation/mutation; L: gene loss or pseudogenization; E: gene expansion; -: adaptive evolution of this gene not mentioned in the studies discussed.

Adaptive Evolution in Visual Systems “in Light of” Varying Lighting Conditions			
**Abbreviation**	**Full Name**	**Gene Ontology**	**Mechanisms**
CYP27C1	Cytochrome P450 Family 27	Cytochrome enzyme that catalyzes desaturation from Vitamin A1, converting it to Vitamin A2.	D
GRK1	G-Protein-Coupled Receptor Kinase	G-Protein receptor kinase, phosphorylates rhodopsin and initiates deactivation.	S
LUM	Lumican	Proteoglycan that, in retinae, regulates organization of collagen fibers.	S
LWS	Long-Wavelength-Sensitive Opsin	G-protein-coupled receptor involved in the phototransduction process, sensitive to “reds” and “greens” between 501 and 573 nm.	S
RH1	Rhodopsin	G-protein-coupled receptor involved in the phototransduction process expressed in rods, absorbs in 447–525 nm.	S, L
RH2	Rhodopsin	G-protein-coupled receptor involved in the phototransduction process expressed in rods, absorbs in 452–537 nm.	D, S, L
RHO	Rhodopsin	G-protein-coupled receptor involved in the phototransduction activity.	D
SWS1	Short-Wavelength-Sensitive Opsin	G-protein-coupled receptor involved in the phototransduction process, sensitive to “violet”, including ultraviolet between 347 and 383 nm.	D, L
SWS2	Short-Wavelength-Sensitive Opsin 2	G-protein-coupled receptor involved in the phototransduction process, sensitive to “blue-violet” between 397 and 482 nm.	D, S, L
VHA	Vacuolar-Type H+ -ATPase	Mitochondrial protein that participates in the excretion of H+ from endothelial cells into the lumen.	S
Adaptations for colonization of aquatic and terrestrial environments			
ACAN	Aggrecan	Integral part of extracellular membrane within cartilaginous tissue.	S
AMPD3	Adenosine Monophosphate Deaminase 3	Highly regulated enzyme that hydrolytically deaminates adenosine monophosphate into inosine monophosphate within the adenylate catabolic pathway.	L
ATP8	Mitochondrially Encoded ATP Synthetase Membrane Subunit 8	ATP synthetase that produces ATP from ADP when a protein gradient is present.	S
CALHM1	Calcium Homeostasis Modulator 1	Calcium–ion channel involved in sweet, bitter, and umami taste transduction.	L
DSC1	Desmocollin 1	Calcium-dependent glycoprotein found primarily in epidermal cells constituting adhesive proteins within desmosome cell–cell junctions.	L
DSG4	Desmocollin 4	Transmembrane component of desmosomes.	L
ELK1	ETS Transcription Factor ELK1	Transcription factor that regulates expression by binding to the promoter of the serum response factor gene.	-
FSHR	Follicle-Stimulating Hormone Receptor	Receptor for the follicle-stimulating hormone and functions in gonad development.	S
GNAT3	G Protein Subunit α Transducin 3	G protein subunit involved in bitter, sweet, and umami taste transduction.	L
GSDMA	Gasdermin A	Precursor protein of a cell-pore-forming protein.	L
*HOX*	Homeobox Domain Genes	Family of transcription factor genes involved in body plan along animal bilateral axis.	-
HOXD11	Homeobox D11	Transcription factor part of a family involved in limb and genital development.	S
HOXD12	Homeobox D12	Transcription factor part of a family involved in limb and genital development.	S
HOXD13	Homeobox D13	Transcription factor part of a family involved in limb and genital development.	S
KRT20	Keratin 20	Intermediate filament conferring structural integrity to epidermal cells.	L
KRT9	Keratin 9	Intermediate filament chain expressed in terminally differentiated epidermal cells.	L
LHX3	LIM Homeobox 3	Transcription factor required for pituitary development and motor neuron specification.	-
LYG1	Lysozyme G1	Lysozyme activity	L
LYG2	Lysozyme G2	Lysozyme activity	
MB	Myoglobin	Iron- and oxygen-binding protein typically present in skeletal muscle tissue.	E
MLL	Lysine Methyltransferase 2A	Currently called KMT2A, co-activator involved in transcriptional regulation of genes during early development and hematopoiesis.	S
MMP12	Matrix Metalloproteinase 12	Involved in extracellular matrix breakdown	L
ORA1	Olfactory Receptor Class-A-Like-Protein-1	Olfactory receptor.	E
PIT-1	POU Class 1 Homeobox 1	Transcription factor involved in regulating expression of multiple pituitary development genes and hormones.	S
SULT6B1	Sulfotransferase Family 6B Member 1	Sulfotransferase.	E
TGM5	Transglutaminase 5	Transglutaminase, enzyme that catalyzes crosslinking between glutamine and lysine residues.	L
Adapting to extreme environmental conditions (i.e., hypoxia, salinity, and low temperatures)			
ABCA12	ATP-Binding-Cassette Subfamily A Member 12	ATP-Binding-Cassette transporter protein.	S
AFGP	Antifreeze Glycoprotein	Proteins that can inhibit growth of ice.	S
ALDOA	Aldolase	Glycolytic enzyme.	S
BMP6	Bone Morphogenetic Protein 6	Ligand of the transforming growth factor β, involved in multiple regulatory processes (e.g., iron homeostasis, fat and bone development, and ovulation).	S
CA	Carbonic Anhydrase	Family of genes of zinc metalloenzymes that catalyze reversible hydration of carbon dioxide.	E
COX1	Cytochrome C Oxidase I	Mitochondrial protein, component of the cytochrome C oxidase.	S
COX3	Cytochrome C Oxidase III	Mitochondrial protein, components of the cytochrome C oxidase.	S
CSAD	Cysteine Sulfinic Acid Decarboxylase	Member of the group 2 decarboxylase.	D
CTH	Cystathionine γ-Lyase	Cytoplasmic enzyme involved in the transculturation pathway.	D
EDA	Ectodysplasin A	Membrane protein thought to be involved in cell–cell signaling during development.	S
ENO	Enolase	Glycolytic enzyme.	S
EPAS1	Endothelial Pas Domain Protein 1	Transcription factor involved in the regulation of genes that are controlled by oxygen.	S
EPO	Erythropoietin	Secreted glycosylated cytokine involved in promoting red blood cell production.	D, S
FBP1	Fructose-Bisphosphatase 1	Glucogenesis regulatory enzyme.	S
FBP2	Fructose-Bisphosphatase 2	Glucogenesis regulatory enzyme.	D
G6PCB	Glucose-6-Phosphatase	Involved in the gluconeogenesis pathway.	D
GDF6	Growth Differentiation Factor 6	Ligand of the transforming growth factor β, involved in regulation of genes associated with formation of some bones, joints, limbs, skull, and axial skeleton.	S
GLA	Galactosidase α	Glycoprotein involved in termina hydrolyses from glycolipids and glycoproteins.	S
GPI	Glucose-6-Phosphate Isomerase	Multiple functions; glycolytic enzyme and neurotrophic factor that promote survival of skeletal motor neurons and sensory neurons intracellularly and extracellularly, respectively.	S
GST	Gluthathione-S-transferase	Conjugation of reduced glutathionone, important in detoxification.	E
HB	Hemoglobin	Transport of oxygen.	S
IGFLR1	IGF-Like Family Receptor 1	Possibly a cell membrane receptor for IGF-like proteins.	S
KITLG	KIT Ligand	Ligand of the tyrosine-kinase receptor, a pleiotropic factor involved in embryonic development.	S
LDHA	Lactate Dehydrogenase A	Involved in the pyruvate fermentation to lactate pathway.	S
LDHD	Lactate Dehydrogenase D	Lactate dehydrogenase.	S
NDUFA10	NADH Dehydrogenase 1 α Subcomplex Subunit 10	Subunit of the first enzyme complex in the electron transport chain in the inner mitochondrial membrane.	S
NDUFA9	NADH Dehydrogenase 1 α Subcomplex Subunit 9	Subunit of the first enzyme complex in the electron transport chain in the inner mitochondrial membrane.	S
NDUFAB1	NADH Dehydrogenase 1 α/β Subcomplex 1	Non-catalytic subunit of the NADH complex involved in the mitochondrial inner membrane electron transport.	S
NDUFC2	NADH Ubiquinone Oxidoreductase Subunit C2	Suspected to be non-catalytic, a subunit of the NADH complex involved in the mitochondrial inner membrane electron transport.	S
NDUFV3	NADH Ubiquinone Oxidoreductase Subunit V3	Suspected to be non-catalytic, a subunit of the NADH complex involved in the mitochondrial inner membrane electron transport.	S
NPR1	Natriuretic Peptide Receptor 1	Guanylyl cyclase involved in catalyzing the production of cGMP from GTP.	E
PC	Pyruvate Carboxylase	Involved in glucogenesis, lipogenesis, insulin secretion, and glutamate.	S
PCK1	Phosphoenolpyruvate Carboxykinase 1	Main control point in the glucogenesis.	S
PCXB	Pyruvate Carboxylase	Catalyzes the carboxylation of pyruvate to oxaloacetate a process that requires biotin and ATP.	D
PGC-1	PPARG Coactivator 1 α	Transcriptional coactivator involved in regulation of genes involved in energy metabolism.	S
PITX1	Paired-Like Homeodomain 1	Transcription factor part of a family involved in organ development and bilateral symmetry.	S
PKLR	Pyruvate Kinase L/R	Pyruvate kinase that catalyzes the rate-limiting step in glycolysis.	D
SLC12A3	Solute Carrier Family 12 Member 3	Important in electrolyte homeostasis, cotransporter that mediates sodium and chloride reabsorption.	S
SOD	Sodium Peroxide Dismutase 1	Binds copper and zinc ions. Functions to destroy free radicals in the body.	E
TRPM8	Transient Receptor Potential Cation Channer Subfamily Member 8	Receptor-activated non-selection cation channel, suggested to be involved in low temperature sensation.	S
UCP1	Uncoupling Protein 1	Mitochondrial anion carrier protein.	D
VIPR1	Vasoactive Intestinal Polypeptide Receptor 1	Receptor for the vasoactive intestinal neuropeptide.	S
VHL	Von Hippel–Lindau Tumor Suppressor	Involved in ubiquitination of the hypoxia-inducible factor.	S
VOM1	Vitelline Membrane Outer Layer 1	Vitelline membrane outer layer proteins.	E
ZP	Zona Pellucida Glycoproteins	Proteins involved in the composition and fertilization functions, and preimplantation development in the zona pellucida.	E
Adaptations to dietary changes			
ACAD9	Acyl-CoA Dehydrogenase Family Member 9	Localized in mitochondria and involved in catalyzing the rate limiting step of the β-oxidation of fatty acyl-CoA.	S
AGT	Alanine-Glyoxylate Aminotransferase	Also known as AGXT, involved in glyoxylate detoxification. In carnivores, the AGT is needed in the mitochondria, while in herbivores peroxisomes, and omnivores, have AGT in both organelles.	E
AMY	α-Amylase	Carbohydrase, specifically amylase.	S
AMY1B	Amylase α 1B	Secreted protein that catalyzes the first steps in digestion of starch and glycogen.	L
ASPM	Assembly Factor For Spindle Microtubules	Essential in mitotic spindle function in embryonic neuroblasts.	S
BARX1	BARX Homeobox 1	Homeobox transcription factor, suggested to be involved in teeth and craniofacial mesenchyme of neural crest origin.	S
BMP2	Bone Morphogenetic Protein 2	Ligand of the transforming growth factor β, involved in bone and cartilage development.	S
BMP4	Bone Morphogenetic Protein 4	Ligand of the transforming growth factor β, involved in heart development and adipogenesis.	S
CDK5RAP2	Cyclin-Dependent-Kinase 5 Regulatory Subunit-Associated Protein 2	Plays a role in centriole engagement and microtubule nucleation.	S
CEP152	Centrosomal Protein 152	Thought to play a role in centrosome function.	S
CYP7A1	Cytochrome P450 Family 7 Subfamily A Member 1	Monooxygenase with lipase catalytic function.	S
DGAT2	Diacylglycerol O-Acyltransferase 2	Enzyme involved in catalyzing the final reaction in triglyceride synthesis.	D
DLX2	Distal-Less Homeobox 2	Homeobox transcription factor, postulated to play a role in forebrain and craniofacial development.	S
EDAR	Ectodysplasin A Receptor	Receptor for Ectodyplasin A and can activate multiple cell death pathways.	S
EHHADH	Enoyl-CoA Hydratase	Bifunctional enzyme part of the peroxisomal β-oxidation pathway.	E
EVE1	EVE1	Transcription factor.	S
IFG-1	Insulin Growth Factor 1	Similar to insulin in function and structure, involved in mediating growth and development.	S
IRX5A	Iroquis Homeobox 5A	Transcription factor involved in cell development, embryonic skeletal joint development, chondrocyte differentiation, and neuron differentiation.	L
LHX7	LIM Homeobox 7	Transcription factor with zinc-finger motifs, involved in patterning and differentiation of multiple tissue types.	S
LHX8	LIM Homeobox 8	Transcription factor with zinc-finger motifs, involved in patterning and differentiation of multiple tissue types.	S
LIPF	Lipase F Gastric Type	Gastric lipase involved in digestion of triglycerides.	S
MAO	Monoamine Oxidase A	Enzyme involved in the catalysis of the oxidative deamination of amines of three neurotransmitters (i.e., dopamine, norepinephrine, and serotonin).	S
MCPH1	Microcephalin 1	DNA damage response protein.	L
NOX4	NADPH Oxidase 4	Catalytic subunit of the NADPH oxidase complex.	L
ODAM	Odontogenic Ameloblast-Associated Protein Precursor	Tooth-associated epithelial protein thought to have a role in odontogenesis.	D
PAX9	Paired Box 9	Paired box domain transcription factor with a critical role in fetal development and cancer growth.	S
PGA	Pepsin A	Enzyme that participates in digestion by activating pepsinogen.	S
PITX2	Paired-Like Homeodomain 2	Homeodomain transcription factor, regulates procollagen lysyl hydroxylase gene expression, as well as basal and hormone regulated activity of prolactin. Involved in development of the eye, tooth, and abdominal organs.	S
PLRP2	Pancreatic Lipase-Related Protein 2	Lipase for hydrolyzation of galactolipids.	E
RNASE1	Ribonuclease A Family Member 1 Pancreatic	Pancreatic-type of secretory ribonuclease.	E
RPFA	Resuscitation-Promoting Factor A	Protein with peptidoglycan hydrolytic activity functions as a factor that stimulates resuscitation of dormant cells.	D
RUNX2	RUNX Family Transcription Factor 2	Transcription factor with Runt DNA-binding domain, essential for osteoblastic differentiation and skeletal morphogenesis.	S
SALL1A	Spalt-Like Transcription Factor 1	Transcription factor with zinc-finger motifs.	L
SCN8A	Sodium Voltage-Gated Channel α Subunit 8	Forms the ion-pore region of the voltage-gated sodium channel. Involved in depolarization during the formation of an action potential in excitable neurons.	S
SCN9A	Sodium Voltage-Gated Channel α Subunit 9	Ion channel activity and sodium ion binding.	S
SCNA4A	Sodium Voltage-Gated Channel α Subunit 4	Transmembrane glycoprotein, involved in generation and propagation of action potentials in neurons and muscle.	S
SCPP5	Secretory Calcium-Binding Phosphoprotein 5	Secretory Calcium-Binding Phosphoprotein.	D
SHH	Sonic Hedgehog	Protein in the Sonic Hedgehog signaling pathway, essential in patterning in early embryonic development.	S
SHOX	Short Stature Homeobox	Homeodomain transcription factor	L
SLC2A3	Solute Carrier Family 2 Member 3	Enables dehydroascorbic acid transmembrane transporter activity	D
TAS2R	Taste 2 Receptor	Transmembrane G-protein-coupled receptor involved in ability to taste glucosinolates and bitter compounds in plants.	L
TBX4	T-Box Transcription Factor 4	Transcription factor with a T-Box binding domain. It has been suggested to play a role in limb development.	L
TFAP2A	Transcription Factor AP 2 α	Transcription factor that works both as a suppressor and activator of multiple genes.	S
TST	Thiosulfate Sulfurtransferase	Catalyzes the conversion of thiosulfate and cyanide to thiocyanate and sulfite	D
UNK	Unk Zinc Finger	RNA-binding protein involved in establishment and maintenance of the early morphology of cortical neurons in embryonic development.	D
WDR62	WD Repent Domain 62	Proposed to play a role in cerebral cortical development.	S
Environmental Pathogens			
APOBEC	Apolipoprotein B MRNA Editing Enzyme Catalytic Subunit 3G	Catalyzes site-specific deamination of RNA and single-stranded DNA.	S
CD14	Myeloid Cell-Specific Leucine-Rich	Surface antigen preferentially expressed on monocytes/macrophages.	S
CD22	Sialic Acid-Binding Ig-Like Lectin 2	Carbohydrate binding protein. Mediates B-cell/B-cell interactions. Thought to localize B-cells in lymphoid tissues.	E
CD40	Tumor Necrosis Factor Receptor Superfamily Member 5	Receptor on antigen-presenting cells of the immune system.	S
CD80	T-Lymphocyte Activation Antigen CD80	Membrane receptor activated by CD28 involved in costimulatory signal essential for T-lymphocyte activation.	S
IFNAR2	Interferon α and β Receptor Subunit 2	Type I membrane protein, forms one of the chains of a interferon α and β receptor.	S
IGH	Immunoglobulin Heavy Locus	Antigen-binding protein involved in the Lectin-induced complement pathway and NFAT immune response.	S
IGHE	Immunoglobulin Heavy Constant Epsilon	Antigen-binding protein involved in Interleukin 4-mediated and cytokine signaling.	S
IGHM	Immunoglobulin Heavy Constant Delta	Antigen binding.	S
LY96	Lymphocyte Antigen 96	Confers responsiveness to lipopolysaccharides when it associates with a Toll-like receptor.	S
MHC	Major Histocompatibility Complex class I	Family of genes associated with antigen processing.	E
MDA5	Interferon Induced with Helicase C Domain 1	Intracellular sensor of viral RNA that triggers innate immune response.	S
MUC7	Mucin 7-Secreted	Salivary mucin thought to play a role in facilitating clearance of bacteria in the mouth.	S
MX	MX Dynamin-Like GTPase	Guanosine triphosphate metabolizing protein that participates in antiviral response.	S
NLPRP3	NLR Family Pyrin Domain-Containing 3	Peptidoglycan binding protein.	E
SSC4D	Scavenger Receptor Cysteine-Rich Family Member with 4 Domains	Scavenger receptor activity.	E
TAB 1	TGF β-Activated Kinase 1	Regulator of the MAP kinase kinase kinase. Thus, mediates various intracellular signaling pathways.	S
TICAM1	Toll-Like Receptor Adaptor Molecule 1	Adaptor protein with Toll/Interleukin 1-receptor homology domain. Protein kinase binding and obsolete signal transducer activity.	S
TLR	Toll-Like Receptors	Family of genes that play a fundamental role in pathogen recognition and activation of innate immunity.	-
TLR1	Toll-Like Receptor 1	Non-viral pathogen recognition, protein heterodimerization activity, and transmembrane signaling.	E
TLR2	Toll-Like Receptor 2	Non-viral pathogen recognition, protein heterodimerization activity, and transmembrane signaling.	E, S
TLR4	Toll-Like Receptor 4	Non-viral pathogen recognition, lipopolysaccharide binding.	S
TLR5	Toll-Like Receptor 5	Non-viral pathogen recognition, protein heterodimerization activity, and transmembrane signaling.	S
TLR7	Toll-Like Receptor 7	Transmembrane signaling receptor activity and double-stranded RNA binding.	E, S
TLR8	Toll-Like Receptor 8	RNA binding and drug binding.	E, S
TLR9	Toll-Like Receptor 9	Transmembrane signaling receptor activity and siRNA binding.	E
TLR22	Toll-Like Receptor 22	Transmembrane signaling receptor activity.	E
TLR23	Toll-Like Receptor 23	Receptor activity.	E
TLR25	Toll-Like Receptor 25	Transmembrane signaling receptor activity.	E
Other Factors			
MSX2	Homeobox Protein MSX-2	Transcription factor with a homeobox-binding domain.	E
C6AST	Calpastatin	Endogenous calcium-dependent cysteine protease inhibitor.	E
PASTN	Pasttristacin	Metalloendopeptidase activity.	E
CRISP	Cysteine-Rich Secretory Proteins	Associated with reptilian venom production and mammalian reproduction.	E
PER2	Period Circadian Regulator 2	Transcription factor and “activator” activity. Primary circadian pacemaker in the mammalian brain.	S

## Data Availability

No new data were created in this study. All the papers used in the systematic review are cited in the reference list [3,4,5,6,7,8,9,10,11,12,13,14,15,16,17,18,19,20,21,22,23,24,25,26,27,28,29,30,31,32,33,34,35,36,37,38,39,40,41,42,43,44,45,46,47,48,49,50,51,52,53,54,55,56,57,58,59,60,61,62,63,64,65,66,67,68,69,70,71,72,73,74,75,76,77,78,79,80,81,82,83,84,85,86,87,88,89,90,91,92,93,94,95,96,97,98,99,100,101,102,103,104,105,106,107,108,109,110,111,112,113,114,115,116,117,118,119,120,121,122,123,124,125,126,127,128,129,130,131,132,133,134,135,136,137,138,139,140,141,142,143,144,145,146,147,148,149,150,151,152].
